# *Mycobacterium peregrinum* Pacemaker Pocket Infection: A Case Report and Review of the Literature

**DOI:** 10.1155/2020/8831026

**Published:** 2020-10-09

**Authors:** A. F. Lazo-Vasquez, J. A. Gonzales-Zamora

**Affiliations:** Division of Infectious Diseases, Department of Medicine, University of Miami, Miller School of Medicine, Miami, Florida, USA

## Abstract

*Mycobacterium peregrinum* is a rapidly growing mycobacterium (RGM), subspecies of *Mycobacterium fortuitum* complex, which can cause infections in the skin, surgical sites, and central lines. It has also been associated with implantable devices such as cardiac devices. Our objective is to present an atypical clinical case of *M. peregrinum* infection associated with a cardiac device, review the published literature, and highlight the importance of this type of RGM infection to enhance their therapeutic success. Only two other cases have been reported of *M. peregrinum* infection associated with cardiac devices. Diagnosis and treatment of *Mycobacterium peregrinum* infection can be challenging, and the literature is scarce. Better understanding and further research should be conducted regarding this infection.

## 1. Introduction

Nontuberculous mycobacteria (NTM) are environmentally ubiquitous bacteria which have been associated with infections in both immunocompromised and immunocompetent populations [1–3]. Recently, rapidly growing mycobacteria (RGM) have emerged as important human pathogens that can cause a wide range of clinical syndromes in humans, especially *M. abscessus*, *M. chelonae*, and *M. fortuitum* [4, 5]. *Mycobacterium peregrinum* is one of the RGM that belong to the *Mycobacterium fortuitum* complex and has been rarely reported to infect humans [3]. Overall, the incidence and importance of infections by RGM have been on the rise, and *M. peregrinum* has been associated with central lines, surgical sites, soft tissue, and skin infections [5–7]. Here, we present a case of a permanent pacemaker pocket site infection caused by *M. peregrinum.*

## 2. Case Report

**Figure 1:**
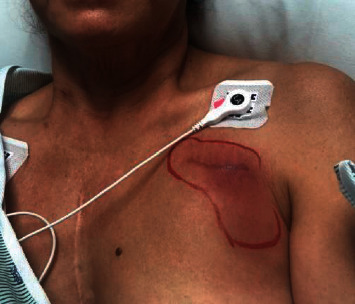
Pacemaker pocket site with developing erythema around surgical scar.

**Figure 2:**
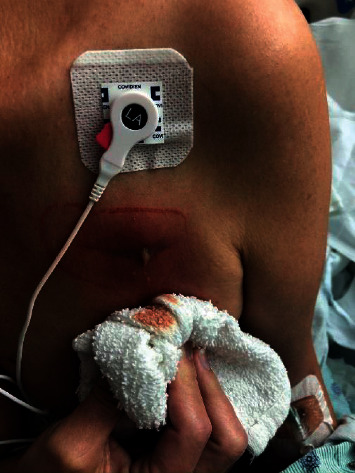
Pacemaker pocket site with purulence expression from the surgical scar.

A 59-year-old woman with a history of recent placement of a dual-chamber permanent pacemaker (PPM) due to sick sinus syndrome was admitted to the hospital due to erythema, warmth, and fluctuance over the PPM site (Figure 1). She was at 4 weeks after PPM placement. Her symptoms developed gradually over a course of 5 days. She denied any fever or chills. She did not have any specific exposures over the PPM area other than dressing changes and cleaning with water and soap. There was no recent travel or pet exposure. Her vital signs on admission showed a temperature of 36.5°C, heart rate of 93 beats per minute, respiratory rate of 18 breaths per minute, and blood pressure of 100/60 mmHg. Physical examination on admission revealed a nondistressed woman, and her chest showed erythema overlying the PPM site with pain to palpation, fluctuance, and evidence of pus from surgical scar (Figure 2). Her laboratory workup showed a white blood cell (WBC) count of 7300/*μ*L, with 80.3% neutrophils, hemoglobin (Hgb) of 12.8 g/dL, platelet count of 189000/*μ*L, lactate of 1.4 mmol/L, glucose of 142 mg/dL, BUN of 9 mg/dL, and creatinine of 0.66 mg/dL.

On the day of admission, blood cultures were obtained, and she was started on broad-spectrum antibiotics with vancomycin 1250 mg IV every 12 hours and piperacillin/tazobactam 4.5 g IV every 8 hours. She then underwent PPM removal on the 2^nd^ day of hospitalization. Dual-chamber leads were taken out, and intraoperative specimens from the pocket site were sent to microbiology for stains and cultures. Pathology samples were not sent except for the leads for gross pathological evaluation. No frank pus was obtained intraoperatively, and the leads were removed without any complications. Blood cultures remained negative, and initial gram stain from the intraoperative specimens showed no organisms and rare WBCs. On day 4 of hospitalization, microbiology reported scant growth of acid-fast bacilli from the deep wound cultures, and empiric therapy was changed to amikacin 10 mg/kg IV daily, imipenem 1 g IV twice daily, and linezolid 600 mg PO twice daily. The specimen was then sent to the National Jewish Health reference laboratory (Denver, Colorado, USA) for identification by rpoB gene sequencing, which showed *Mycobacterium peregrinum*.

The patient showed clinical improvement and was discharged from the hospital on day 5 with the proposed empiric intravenous regimen. At 3 weeks of follow-up, the patient reported no side effects from the antibiotics prescribed and had proper healing of the PPM pocket site. Full susceptibilities by broth microtiter dilution methodology were made available (Table 1), and she was switched to moxifloxacin 400 mg PO daily and trimethoprim/sulfamethoxazole 160/800 mg twice daily to complete a 4-month regimen. Of note, the erm(39) gene was not tested for this case, and it was not preincubated in subinhibitory concentrations of macrolide to check for inducible resistance.

**Figure 3:**
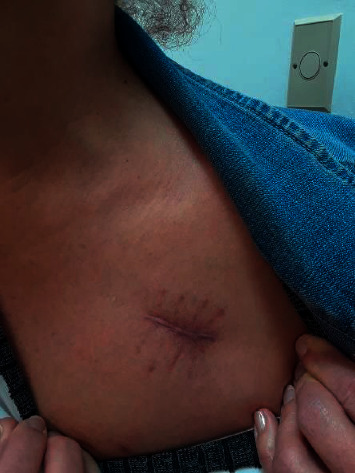
Pacemaker pocket site 2 months after the removal of pacemaker and antibiotic therapy.

The patient was seen at 2 months and completed therapy at 4 months with proper healing of the PPM pocket site (Figure 3). She had no side effects from the antibiotics and did not require reimplantation of the PPM.

## 3. Discussion

*Mycobacterium peregrinum* is an RGM found ubiquitously in the environment that belongs to the *Mycobacterium fortuitum* group [1–3]. The clinical presentation of skin and soft tissue infections from RGM usually manifest as localized cellulitis, abscess, sinus tracts, nodules, or chronic ulcers [8, 9]. It has been postulated that these organisms may preferentially colonize the anterior chest wall since most documented human infections involve the anterior chest such as tunneled catheter infections, sternotomy wound infections, and infections after augmentation mammoplasty procedures [8, 10–12].

There have been several published cases related to *M. peregrinum* causing infections such as surgical site, cardiac devices, soft tissue, and central catheters [3–7, 13–15]. To our knowledge, there have only been three reported cases of *M. peregrinum* infection associated with cardiac devices, including our patient. One case of *M. peregrinum* infection of an automatic implantable cardioverter-defibrillator was described by Short et al. [12] and another case of a pacemaker lead infection by Amraoui et al. [16]. The case by Short et al. described a similar presentation of *M. peregrinum* infection of a recently placed cardiac device. The patient was treated with a regimen of ciprofloxacin and clarithromycin for a total of 6 weeks. The case by Short et al. involved an *M. peregrinum* infection of the leads of a PPM and associated bacteremia, and he was treated with clarithromycin and ciprofloxacin for a few months and cure was achieved only after removal of the device.

Regarding treatment, RGM have a distinctive antimicrobial susceptibility profile depending on the species, and initial antibiotic treatment should be tailored to this. One must also take into consideration the presence of erm genes which confers resistance to macrolides [17]. Most *M. fortuitum* isolates have an erm gene that could be active or inducible, and macrolides should be used with caution [18]. Studies testing for erm(38) and erm(39) resistance genes showed that the subspecies *M. peregrinum* does not contain them, but it can still have variable MICs to macrolides due to other mechanisms [19].

For definite treatment, identifying the organism to the species level is paramount to ensure proper antibiotic selection necessary to overcome this infection [6]. Removal of the infected device is also essential to guarantee the best chance at cure [18]. Treatment guidelines published jointly by the American Thoracic Society and the Infectious Diseases Society of America (ATS/IDSA) are a valuable resource, but treatment should be tailored to the susceptibility profile of the species. *M. peregrinum*, a subspecies of the *M. fortuitum* complex, is usually susceptible to multiple oral antimicrobial agents, including newer macrolides and quinolones, doxycycline, minocycline, and sulfonamides [18]. At least two agents should be utilized and then tailor the therapy to the susceptibility profile once obtained as they did on both cases reviewed here [12, 16].

The duration of antibiotic treatment for this specific type of infection is variable. The IDSA guidelines suggest for serious soft tissue infections with *M. fortuitum* species to treat for at least 4 months [18]. A review of 11 surgical site infections with *M. peregrinum* by Nagao et al. reported a duration ranging from 6 weeks to 4 months [6]. The two cases of *M. peregrinum* infection associated with cardiac devices mentioned here are based on their treatment duration on clinical response and patient tolerability. One case treated the patient for 6 weeks [12]. The other case treated the patient for several months without success until the cardiac device was completely removed, achieving cure [16]. We treated our case for a total of 4 months and obtained excellent outcomes.

## 4. Conclusions

This case highlights the clinical presentation of an NTM infection related to cardiac devices, a complication that is rare and difficult to treat. To our knowledge, this is the third reported case of *M. peregrinum*, a subspecies of *M. fortuitum* group, causing this type of infection. Diagnosis and treatment are challenging, and therefore, having a high degree of suspicion is key. In the case of culture-negative infection of a cardiac device or around the pocket site, physicians should consider the need for extended incubation or dedicated mycobacterial cultures to improve the yield of NTM growth, especially with the rising significance of various NTM infections. Treatment recommendations for this specific site of infection are limited to expert opinion, but guidelines can be extrapolated from soft and skin tissue infections recommendations of *M. fortuitum*. Combination therapy with at least two agents is necessary to avoid resistance and should be given for at least 4 months. Removal of the infected device is recommended and seems to be essential for cure. Further research is required to elaborate a comprehensive and protocolized management strategy for these infections.

## Figures and Tables

**Table 1:** Antibiotic susceptibility and interpretation of *M. peregrinum* isolates.

Antibiotics	MIC (mcg/mL)	Interpretation
Amikacin	≤8	*S*
Augmentin	32/16	TR
Azithromycin	≤16	TS
Cefepime	>32	TR
Cefotaxime	>64	TR
Cefoxitin	≤16	*S*
Ceftriaxone	>64	TR
Ciprofloxacin	≤1	*S*
Clarithromycin	≤0.25	*S*
Clofazimine	≤0.5	TS
Clofazimine/amikacin	≤0.5/2	TS
Doxycycline	8	*R*
Gentamicin	≤2	TS
Imipenem	≤2	*S*
Kanamycin	64	TR
Linezolid	≤1	*S*
Minocycline	≤1	TS
Moxifloxacin	≤0.5	*S*
Tigecycline	≤0.25	TS
Tobramycin	16	*R*
Trimethoprim/sulfamethoxazole	≤0.5/9.5	*S*

*S* = susceptible. *I* = intermediate. *R* = resistant. NI = no CLSI interpretive interpretative guidance. TS = tentative interpretation susceptible. TI = tentative interpretation intermediate. TR = tentative interpretation resistant.

## Data Availability

The data used to support the findings of this study are included within the article.
